# A data-driven statistical model that estimates measurement uncertainty improves interpretation of ADC reproducibility: a multi-site study of liver metastases

**DOI:** 10.1038/s41598-017-14625-0

**Published:** 2017-10-26

**Authors:** Ryan Pathak, Hossein Ragheb, Neil A. Thacker, David M. Morris, Houshang Amiri, Joost Kuijer, Nandita M. deSouza, Arend Heerschap, Alan Jackson

**Affiliations:** 1 University of Manchester, Wolfson Molecular Imaging Centre, Manchester, UK; 2 Radboudumc, Radiology and Nuclear Medicine, Nijmegen, Gelderland, NL Netherlands; 3 VU University Medical Center, Physics & Medical Technology, PO Box 7057, Amsterdam, NL 1007MB Netherlands; 4 Institute of Cancer Research, MRI Unit, Downs Road, Sutton, Surrey, SM2 5PT UK; 5 Neuroscience Research Center, Institute of Neuropharmacology, Kerman University of Medical Sciences, Kerman, Iran

## Abstract

Apparent Diffusion Coefficient (ADC) is a potential quantitative imaging biomarker for tumour cell density and is widely used to detect early treatment changes in cancer therapy. We propose a strategy to improve confidence in the interpretation of measured changes in ADC using a data-driven model that describes sources of measurement error. Observed ADC is then standardised against this estimation of uncertainty for any given measurement. 20 patients were recruited prospectively and equitably across 4 sites, and scanned twice (test-retest) within 7 days. Repeatability measurements of defined regions (ROIs) of tumour and normal tissue were quantified as percentage change in mean ADC (test vs. re-test) and then standardised against an estimation of uncertainty. Multi-site reproducibility, (quantified as width of the 95% confidence bound between the lower confidence interval and higher confidence interval for all repeatability measurements), was compared before and after standardisation to the model. The 95% confidence interval width used to determine a statistically significant change reduced from 21.1 to 2.7% after standardisation. Small tumour volumes and respiratory motion were found to be important contributors to poor reproducibility. A look up chart has been provided for investigators who would like to estimate uncertainty from statistical error on individual ADC measurements.

## Introduction

Diffusion weighted imaging (DWI) is a Magnetic Resonance Imaging (MRI) sequence acquisition that is sensitive to free water diffusion^1,2^. Regions of reduced extra-cellular space due to high cell density or other micro environmental factors will result in restricted diffusion of free water relative to surrounding tissue. Similarly, increases in extravascular-extracellular space due to cell death may result in increased free water diffusion. Consequently, apparent diffusional coefficient (ADC) derived from diffusion weighted MRI has received considerable attention as a potential biomarker of early response to cytotoxic therapies^3^. The ADC is the decay constant, calculated from 2 or more DWI images, acquired with increasing sensitivity to water mobility. A high ADC corresponds to increased water mobility towards free diffusion, and conversely a low ADC corresponds to restricted diffusion. In a densely cellular homogeneous tumour, such as lymphoma, treatment-related ADC changes may be as high as 50%^4^, however treatment responses may be heterogeneous due to regional micro environmental factors or genetic variation^5–7^. A recent animal model study of ovarian tumours showed an average 7.5% increase in mean ADC after treatment but identified significant spatial heterogeneity due to variations in tumour response^7^.

In therapeutic studies using ADC early treatment induced changes are typically in the range of 10–30%^8,9^. Statistically, in order to detect a 10% change in mean ADC for an individual lesion, with 95% reliability, a test-retest repeatability of 3–4% is required (assuming that the distribution of ADC measures is Gaussian). If repeatability is worse than this, then our ability to detect true biological change of this magnitude is lost. A repeatability of 3% may be difficult to achieve, particularly in multi-site, multi-vendor trials, although studies in phantoms and homogenous healthy liver taken across multiple sites, have shown repeatability of 1–4% and 3–7% respectively^10^. To our knowledge there is no published data to describe ADC reproducibility of liver metastases in a multi-site, multi-vendor setting.

Factors that negatively affect repeatability relate to the tumour itself (size^11^, heterogeneity^12^ and site^13^), image quality (signal to noise ratio (SNR)^14^, motion^15^), curve-fitting techniques^16^ and errors related to the MR system^17^. Voxel-wise quantitative DWI in the liver is also specifically degraded by respiratory motion artefact, with little improvement and mixed results when using on-table compensation methods such as navigator echo and respiratory gating^18–20^. Consequently, a change in ADC due to measurement errors may be interpreted as disease progression or response where response thresholds are derived from group-wise reproducibility data.

The primary endpoint of this study was to define a statistical model of predictable sources of variability that contribute to measurement error, and fit this to observed data in order to quantify the level of uncertainty in mean ADC repeatability. Through standardisation of repeatability measurements for predictable sources of statistical variability that contribute to uncertainty in the mean ADC, we sought to increase our confidence in detecting genuine post treatment changes for future studies. We have conducted this study in patients with colorectal liver metastases, which is a commonly studied pathology in novel therapeutic trials. There is considerable difference in the appearance and margination of metastases from different primary tumours and the potential impact of this will be discussed below.

## Materials and Methods

### Patients

This multi-site prospective study was compliant with; 1) Medical Research Involving Human Subjects Act (WMO) and approved by a certified Medical Ethics Committee (MEC) institutional review board at The VU University Medical Centre, Amsterdam, and Radboud University Nijmegen Medical Centre. 2) Compliant with and approved by the NHS Health Research Authority Research Ethics Committee, United Kingdom, following approval from local Research & Development administrations at The Christie Hospital NHS Trust, Manchester, and The Royal Marsden Hospital NHS Trust, London. Formal written informed consent was recorded for each volunteer that participated. Inclusion criteria included; Histological diagnosis of primary colorectal carcinoma, radiological evidence of at least one liver metastasis (minimum volume 2 cm^3^), new diagnosis or no ongoing treatment. Exclusion criteria included; Contraindication to MRI, ongoing treatment. Patients who met the inclusion criteria were scanned consecutively, as and when they appeared at their respective oncology clinic, prior to any new treatment commencing.

### Image acquisition

Patients were imaged twice within 7 days, using 1.5T MR systems from 3 vendors (Table 1). DWI parameters were as follows; b value images for 3 orthogonal gradient directions, 4 signal averages per image, free-breathing single shot echo-planar sequence (SS-EPI), spectral attenuated inversion recovery (SPAIR) fat suppression, 5 mm axial slice thickness, 40 slices with no inter-slice gap, target FOV of 380 mm (380 × 380 for GE, 384 × 384 for Philips and 332 × 380 for Siemens), bandwidth 1400–1800 Hz per pixel, pixel size of 1.5 × 1.5 mm, acquisition matrix 128 × 112 or 128 × 128.

**Table 1:** List of MR systems and receiver coils used, with variable DWI acquisition parameters.

MRI (1.5 T)	Body coil	Parallel imaging	B-values (s/mm^2^)	TR/TE (ms)
Siemens Magnetom Avanto	6 channel	GRAPPA 2	100, 500, 900	8000/76
General Electric (GE) Signa HDxt	8 channel	ASSET	100, 500, 900	8500/74
Philips Achieva	8 channel	SENSE	0, 100, 500, 900	8000/88

### Image analysis

**Figure 1:**
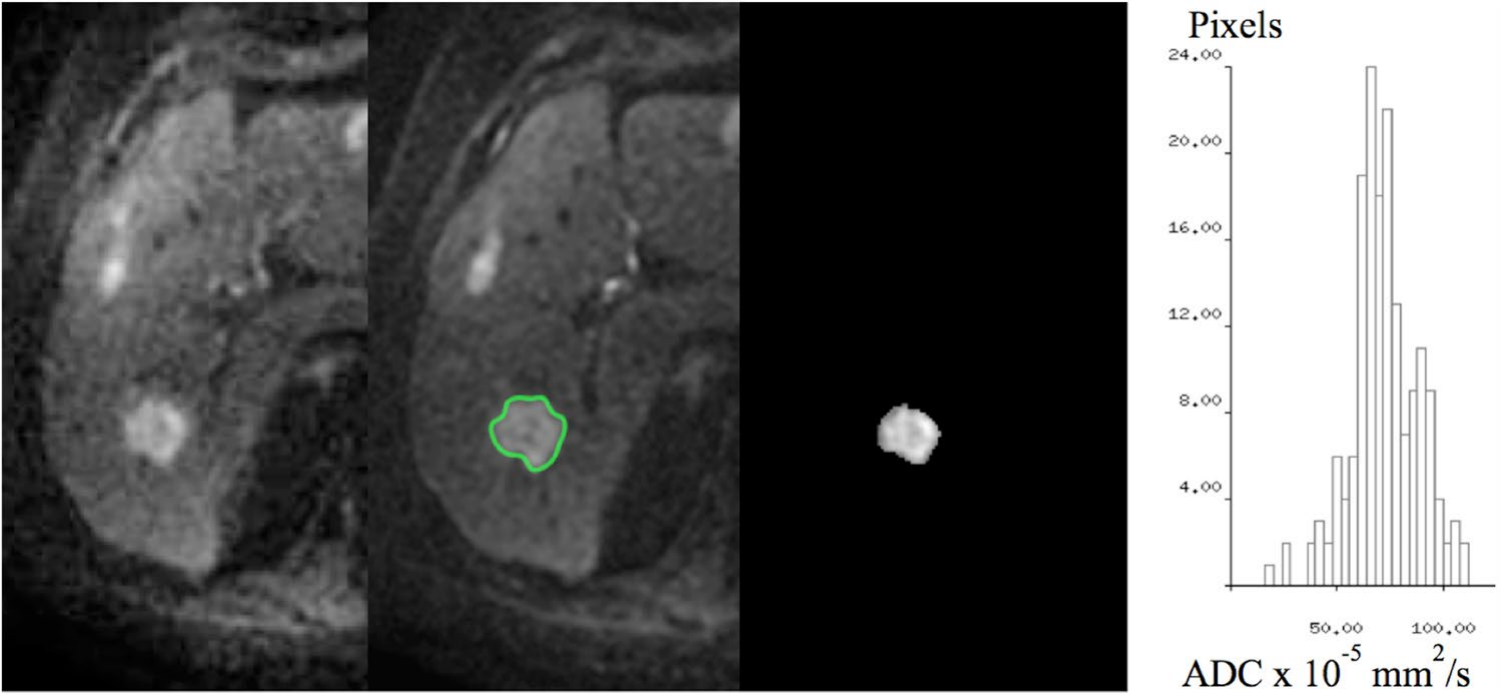
Tumour selection and image analysis. A single lesion is chosen based on size and location from b-100 DWI images (right image) and a ROI is manually defined for each test-retest data set (middle image). A parametric map of ADC values is calculated for each pixel within the ROI (left image). For 3D volumes, the voxel ADC values within each slice ROI is combined and represented as a histogram (far left).

A single lesion was chosen based on size (the largest visible single tumour or indistinguishable tumour conglomerate with a continuous circumference that was ≥2 cm^3^) and location (right lobe, away from the heart or diaphragm where possible). A single observer manually outlined whole tumour 3-dimensional (3D) regions of interest (ROI) from b-100 images for each test-retest measurement (Osirix v.5.8.2 32 bit viewing software) (Fig. 1). The first and last MRI slices through the tumour were excluded to minimise partial volume effects, whereby voxels at the edge of tumours contain both abnormal and normal tissue, resulting in artefactual reduction of tumour signal intensity. Automated or semi-automated selections of ROIs are widely used for areas of the body where movement artefact is less problematic, such as the brain^21^. Improvement in interobserver agreement from semi-automated methods can also be achieved in the liver^22^ however any reduction in the quality of acquisition (such as low SNR or motion artefact) will be associated with problems related to identification of the boundaries of the lesion. In patients with tumours that are highly heterogeneous automated methods may select only areas above a chosen threshold. Identification of outer boundaries from motion-affected tumours can also be problematic. Since our intention was to provide a proof of concept for the benefits of error modelling we have chosen to use manual delineation of tumour volumes since this is the typical method used in the majority of studies of ADC in the liver^2,23^.

In order to develop and test the proposed error model (see below) we wished to maximise the range of ROI sizes available. Consequently, 2 additional single slice ROIs were defined within the delineated 3D tumour volume; 1) a slice representing the largest area through the lesion and 2) a slice that best represented mostly solid tumour. This reflects common practice in previous studies, for example where ADC metrics have been calculated from ROIs based on a single slice with the largest diameter^24,25^, or occasionally a prescribed 2D area believed to be solid tumour^26^. We could expect a single observer definition of the largest diameter slice within a tumour to be fairly robust, however the largest diameter slice normally contains the most central necrotic and cystic tissue. Although more subjective, a slice with the most solid tumour may be a better representation of cell density. It is important that we emphasise at this point, the primary purpose for defining further 2D small volume ROIs was to increase the accuracy of fit to our error model, and explore the relationship between statistical error and ROI size. Studies comparing 2D axial ROIs and prescribed ROIs, to 3D volumes^11,22^, have found whole tumour volumes to be more reproducible. Published consensus guidelines for diffusion imaging recommend 3D volumes^27^.

In addition, ROIs of a fixed dimension were defined over normal appearing liver parenchyma away from obvious tumour.

Voxel ADC values were estimated from the mono-exponential fit of 3 b-value images (100, 500, 900 s/mm^2^) corrected for high b-value SNR bias^28^ (see Supplementary information appendix 1). A frequency distribution histogram of ADC values within each ROI was generated, and a mean ADC for the whole ROI calculated (Fig. 1).

### Statistical model of expected measurement error

The sample size chosen for this study, split equally between sites, is comparable to previous studies^2,23^. We have chosen to use percentage change in ADC (ΔADC%), which provides a metric of repeatability for individual tumour measurements that can be directly compared within and between studies^29^. This is an ideal repeatability metric for monitoring post treatment changes for individual patients. For comparison between studies, the 95% confidence interval width can be used to define statistically significant change in ADC measurements.

Assuming a Gaussian distribution of ADC the accuracy of any data-driven estimate of the mean value or of the distribution width will become more accurate as the sample size increases. Consequently, sample size in voxels (equivalent to tumour volume) would be expected to significantly influence the uncertainty in the measurement of mean ADC. Test-retest repeatability, expressed as individual tumour ΔADC% was therefore plotted against tumour volume expressed as the number of voxels in the tumour (log scale) to assess the relationship if any between tumour size and the sources of variation in repeated measures defined by our model (which contribute to measurement uncertainty). A single voxel volume, using the imaging protocol employed here is equivalent to 11.25 mm^3^.

ΔADC%, (the percentage change in ADC between baselines, expressed as *R*_12_, provides a direct measure of repeatability between scans 1 and 2 and is calculated as

1.1 $${R}_{12}=2\frac{({D}_{1}-{D}_{2})}{({D}_{1}+{D}_{2})}\times 100$$

where *D*_1_ and *D*_2_ are test and retest mean ADC values, respectively.

A proportion (${\varepsilon }_{{R}_{12}}$) of the measured repeatability between *D*_1_ and *D*_2_ is due to predictable statistical measurement errors on *D*_1_ and *D*_2_, (${\sigma }_{{D}_{1}}$and ${\sigma }_{{D}_{2}}$respectively). The term ${\varepsilon }_{{R}_{12}}$can be thought of as a measure of the uncertainty of the repeatability measurement and is estimated from error propagation of ${\sigma }_{{D}_{1}}$and ${\sigma }_{{D}_{2}}$ using the equation below (see Supplementary information appendix 2 for derivation)

1.2 $${\varepsilon }_{{R}_{12}}({\sigma }_{{D}_{1}},{\sigma }_{{D}_{2}})=\frac{400\sqrt{{D}_{1}^{2}{\sigma }_{{D}_{2}}^{2}+{D}_{2}^{2}{\sigma }_{{D}_{1}}^{2}}}{{({D}_{1}+{D}_{2})}^{2}}$$

The term ${\varepsilon }_{{R}_{12}}$ is dependent on the measurement accuracy of both the test and retest mean ADCs (${\sigma }_{{D}_{1}}$, ${\sigma }_{{D}_{2}}$). Three parameters were defined within a model to describe ${\varepsilon }_{{R}_{12}}$of the observed repeatability measurement. The simplest assumption is that ${\varepsilon }_{{R}_{12}}$is due only to accumulation of systematic errors related to the MRI scanner. Systematic errors (${\varepsilon }_{sys}$) contribute a fixed proportional error reflecting inability to accurately replicate equivalent image data on repeated attempts. Another possible source of measurement error reflects accuracy of the fitting routine used to estimate voxel ADC values; therefore a second parameter in our model assumes that these are fixed between *D*_1_ and *D*_2_. This is described as a fixed fitting error (${\sigma }_{fix}$). The third parameter takes into consideration the ADC histogram distribution width for *D*_1_ and *D*_2_. This is a measure of the accuracy of the calculated mean A*D*C (*D*_1_, *D*_2_). The standard error is the ratio between the standard deviation of the mean ADC, and the square root of the number of voxels within the ROI. The wider the distribution, the larger the standard error of the mean will be and conversely, the larger the sample size the smaller the standard error of the mean will be (hence the assumption earlier that ROI size is an important variable for repeatability). In addition to these factors, we would also expect ADC distribution width, and therefore mean ADC measurement accuracy, to be affected by SNR and tumour heterogeneity.

In summary, the 3-parameter model of statistical measurement errors include a fixed fitting error term (${\sigma }_{fix}$), a term (*β*) proportional to ADC width and the systematic error (${\varepsilon }_{sys}$), as described in the following equation

1.3 $${\varepsilon }_{{R}_{12}}^{2}={\beta }^{2}{\varepsilon }_{{R}_{12}}^{2}({\sigma }_{{D}_{1}},{\sigma }_{{D}_{2}})+{\varepsilon }_{{R}_{12}}^{2}({\sigma }_{fix},{\sigma }_{fix})+{\varepsilon }_{sys}^{2}$$

A maximum likelihood expectation (MLE) routine was used to fit this general model to the defined 3D and 2D single slice ROIs in order to identify the parameter(s) most predictive of the repeatability measurements obtained (refer to Supplementary information appendix 3). The observed ΔADC% was standardised to ${\varepsilon }_{{R}_{12}}$in order to produce an estimate of reproducibility for the entire group (95% confidence interval widths). In other words, the level of uncertainty in the repeated measures for each ROI was taken into consideration. The parameters (*β*, σ_*fix*_ and ε_*sys*_) that produced the best fit of the data were used to generate a look-up chart for estimating the relationship between ${\varepsilon }_{{R}_{12}}$and the ADC histogram width, for a range of ROI sizes.

Datasets identified as having visible motion artefact were excluded from the MLE model fitting routine as we hypothesise that respiratory motion is an important additional variable affecting reproducibility, independently from the model. Once the best-fit parameters were obtained, all data including those with visible motion were included to compare the reference standard to the index test (data standardised to the level of uncertainty in each observed ΔADC%). A chi-squared goodness of fit method was applied to test the suitability of the error model as a fit for the observed data.

### Data availability

The full dataset of mean and median ADC values calculated from all the ROIs defined for this study, are freely available within the following document:http://www.tina-vision.net/docs/memos/2014-007.pdf

## Results

Twenty patients (5 per site) were scanned between May 2012 and October 2014 (16 males, 4 females; median age 63 years; range 44–77 years). 5 patient data sets (25%) were identified with visible motion in test, retest or both acquisitions. Table 2 is a summary of the following; test-retest average tumour size (voxels), average absolute mean ADC values, the percentage change in tumour size (ΔVOL%) and ADC (ΔADC%) for each patient.

**Table 2:** The ADC values, lesion size and image characteristics for each patient. For 3D whole tumour volumes, the average (*) of two baselines is displayed for; number of voxels (where each voxel is 11.25 mm^3^), mean ADC values (×10^−5^ mm^2^/s). The percentage change in tumour volume and mean ADC between test-retest is given (ΔVOL%, ΔADC%). The data sets visually affected by “Motion” artefact are indicated in the Image column.

Patient	Voxels*	ΔVOL%	ADC*	ΔADC%	Lesion	Image
1	1141	0.44	76	18.56		Motion
2	3214	8.47	102	−22.37	Sub-phrenic	Motion
3	2845	0.21	97	1.17	5% cystic	
4	1297	0.15	77	14.69		
5	603	4.81	98	−3.39	Sub-phrenic	Motion
6	573	2.44	87	−2.52		
7	148	−14.63	123	2.25		
8	3178	−1.48	102	7.60	Sub-phrenic	Motion
9	4589	1.44	95	−4.35		
10	3731	28.19	103	1.69		
11	5957	0.32	140	6.48		
12	74572	−6.04	102	−12.13		Motion
13	6780	−4.09	93	1.22		
14	270	−5.57	93	−7.36		
15	61130	−5.01	118	−1.11		
16	8788	4.79	127	−5.30	10% cystic	
17	4315	−4.82	98	1.06		
18	2140	−2.38	129	1.04		
19	7914	8.38	198	2.84	95% cystic	
20	4304	−3.53	110	−0.31		

The average whole tumour mean ADC was 109 × 10^−5^ mm^2^/s (range 76–198 × 10^−5^ mm^2^/s).

**Figure 2:**
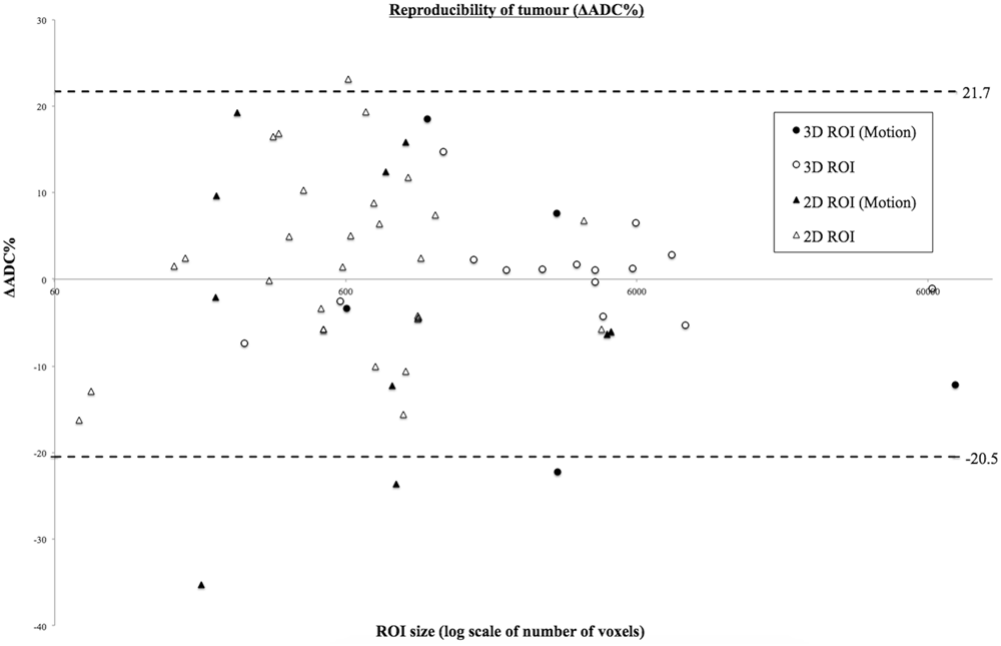
Tumour reproducibility of ΔADC% as measured by the 95% confidence interval width for all multisite data. ∆ADC% is plotted against ROI size (log number of voxels) for 3D and 2D tumour regions (3D circles, 2D triangles). Data affected by motion is highlighted (solid black). The fixed-sized normal parenchyma ROIs are included in the calculation of the 95% CI width of 21.1%.

The observed ΔADC% for each test-retest dataset is plotted against ROI size in Fig. 2. There is a trend in the scatter to suggest repeatability improves with increasing ROI volume, but the overall 95% confidence limit width for all data is 21.1%. The ROI volumes delineated between test-retest data are relatively stable (Table 2), with a mean ΔVOL% of 0.6% (SD 8.2%). The 95% confidence limit width for ΔVOL% is 16.1%. When 2 extreme outliers are removed, this becomes 8.2% for the remaining 18 test retest datasets.

### Applying the 3-parameter model

The contribution of predictable statistical errors to each ΔADC% was estimated using the 3-parameter model described above. The parameters (scaling factors) in the error model were found to be:

$$\beta =4.87,{\sigma }_{fix}=69.35\,and\,{\varepsilon }_{sys}=2.65$$

In the majority of cases *β* had a larger contribution to the measurement error than σ_*fix*_. In most cases was minimal. When *σ*_*fix*_ was removed (i.e. a 2 parameter model), *β* = 5.48 and ε_*sys*_ = 3.89.

The suitability of the error model (i.e. a null hypothesis that the model describes the data accurately) was tested using the Chi-squared (*χ* ^2^) method (see appendix 3 of the Supplementary information). For 3D tumour ROIs, the *χ* ^2^ = 11.33 with 15 degrees of freedom (Probability that the null hypothesis is accepted = 0.73). As there was no significant difference between our model and the observed data (p > 0.05), the null hypothesis was accepted. For the 2-parameter model, for 3D ROIs = 8.79, (p = 0.89) which was marginally worse than when ${\sigma }_{fix}$ had been included. When only using ε_sys_ (i.e. assuming a conventional form where measurement error is simply constant across samples) the model was rejected (p = 0.03).

**Figure 3:**
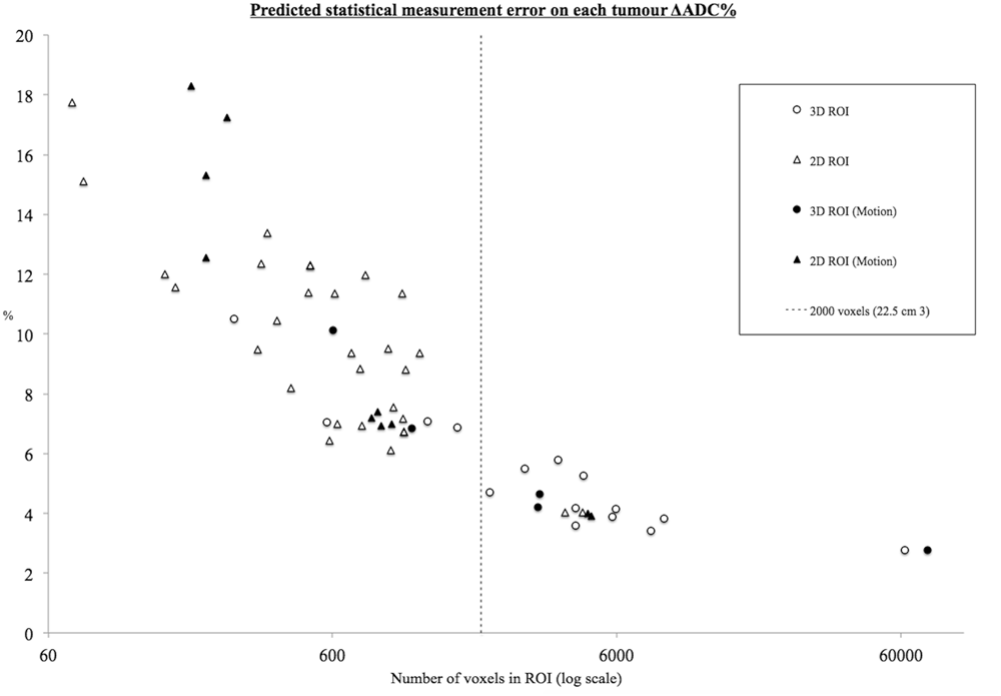
The relationship between statistical measurement error and tumour ROI size. Measurement error improves with increasing ROI size, up to a threshold of around 2000 voxels equivalent to 22.5 cm^3^.

The relationship between the product of the 3-parameter model, ε_*R*12_, and ROI size was plotted for each data set (Fig. 3). There is a clear inverse relationship between expected statistical error and ROI volume. The ε_*R*12_ improved, despite motion, as tumour size increased. Above a threshold value of approximately 22.5 cm^3^ (dashed line in Fig. 3), the rate of improvement began to plateau.

**Figure 4:**
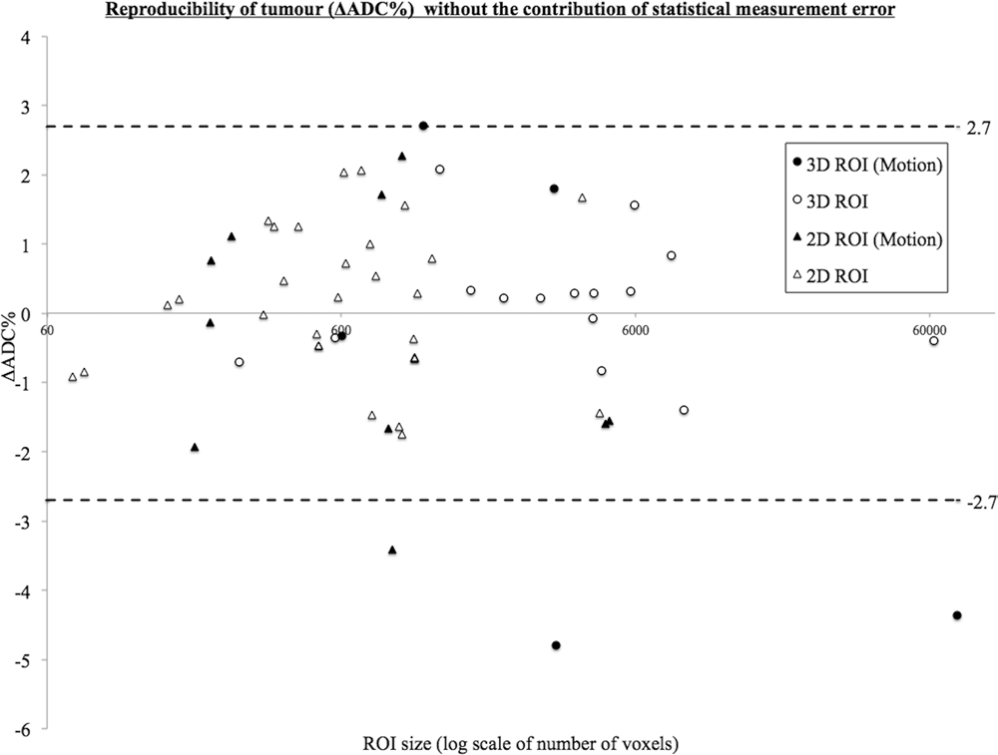
The improvement in estimating repeatability measurements after accounting for the contribution of statistical measurement error. ∆ADC% is plotted against ROI size (log scale of number of voxels) for 3D and 2D tumour regions (3D circles, 2D triangles). Data affected by motion is highlighted (solid black). When the contribution of statistical measurement error is factored out (compared to Fig. 2), the 95% confidence interval width improves from 21.1% to 2.7%. The majority of data affected by motion become outliers, regardless of their size.

When ΔADC% is standardized to its corresponding estimated statistical measurement error, i.e. factoring out the differences in the contribution of statistical measurement error on each ΔADC% (Fig. 4), the 95% confidence interval width used to determine a statistically significant change in ΔADC% reduced from 21.1 to 2.7%. The majority of data affected by gross motion become outliers, regardless of their size.

Using the 3-parameter approach, when the ΔADC% for each ROI used to fit the model, is standardised to its level of uncertainty, the *χ* ^2^ distribution is 59.95 with 60 degrees of freedom (Probability (*χ* ^2^) ≤ 59.95 = 0.48), i.e. there was no significant difference between the standardised distribution and our model, and the data was a good fit. When grouping all tumour ROIs together, *χ* ^2^ istribution is 132 with 15 degrees of freedom (Probability *χ* ^2^ ≤ 132 = 2.4852e–7), therefore the model is rejected. This is to be expected, as data sets with motion artefact are included. When grouping only those tumour ROIs without visible motion, *χ* ^2^ distribution is 46 with 45 degrees of freedom (Probability (*χ* ^2^) ≤ 46 = 0.43), and the model is once more a good fit.

**Figure 5:**
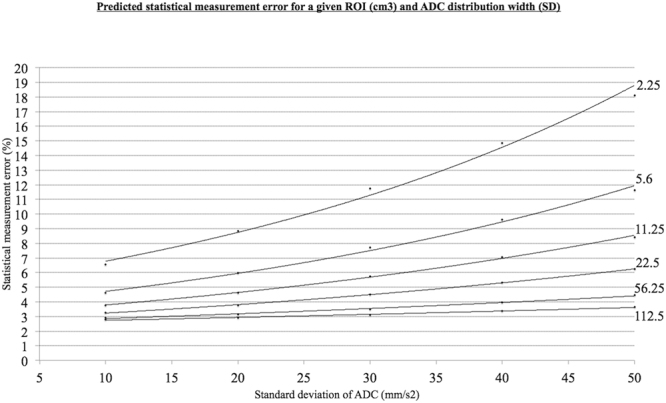
Look up chart for estimating statistical error. Using the parameters that produced the best fit of data, a look-up chart has been created, that can be utilised to estimate statistical measurement error for any ROI with a known ADC histogram width (SD) and size (voxels).

In Fig. 5 a look up chart is presented that can be used to estimate ε_*R*12_ for any ROI with a known ADC histogram width (SD) and size (voxels). This was developed using the parameters ($\beta$, ${\sigma }_{fix}$and ${\varepsilon }_{sys}$) that produced the best fit of data. For example, if an investigator measures ΔADC% of a tumour after treatment to be 25%, and the tumour volume is between 10 and 20 cm^3^, the uncertainty in that estimation of ΔADC% will be approximately between 6 and 18%. This can be quantified more accurately by knowing the ADC distribution width for the ROI histogram. If the standard deviation of the ADC distribution is large e.g. 50 mm/s^2^, then uncertainty is the measurement is around 18%. In comparison, if there is a narrower ADC distribution width for a tumour volume, e.g. 10 mm/s^2^, then the investigator can have more confidence in the ΔADC% measurement of 25% after treatment (approximately 6% uncertainty in the measurement).

In summary, for a small tumour volume, with a wide ADC range of distribution, a higher threshold is required in the interpretation of ΔADC%, in order to overcome uncertainty in the measurement.

## Discussion

Mean ADC is a potential MR imaging biomarker for use in assessment of early treatment response of colorectal liver metastasis^3^. In therapeutic trials early treatment typically induce ADC changes in the range of 10–30%^8,9^. As discussed in the introduction, in order to reliably detect a 10% change in a single lesion requires an accuracy of ADC measurements sufficient to produce test-retest repeatability of 3–4%. In this prospective multi-site, multi-vendor study ΔADC% reproducibility for all tumour ROIs was 21.1% (95% confidence interval width). This compares slightly favourably but to a similar degree, to a previous study that found limits of agreement between 28.7 and 31.3% for short-term reproducibility^30^. For completion, Coefficient of Variance (CoV) was calculated using absolute mean ADC values. A multi-site CoV of 5.3% was comparable to previous single site studies that have measured reproducibility in healthy liver^10,15,31–35^, or liver tumours^30,34,36,37^ using 1.5T or 3T scanners with a variety of protocols and gating methods. The average whole tumour, mean ADC values from this study of 109 × 10^−5^ mm^2^/s (range 76–198 × 10^−5^ mm^2^/s) agree closely with those previously published for colorectal metastases^2,23^.

Any estimate of ADC will be subject to uncertainty from a variety of sources. It is clear from our observations that motion is one of the major sources of error in ADC measurements. This work did not address the movement issue since movement effects cannot be modeled as a fixed error in the parametric images. Another potential source of error is the choice of delineation methods used for identification of ROIs. In this paper we have used manual delineation since is the most commonly employed^2,23^ and has been recommended in consensus reviews^27^. Automated methods which may provide improved accuracy have been widely employed in tissue not subject physiological motion, particularly in studies in the brain^21^. To date, studies of liver tumours have not widely employed automated methods and would require the highest quality images to accurately delineate tumour boundaries. In the current study the average percentage change in tumour volume measurements between baselines was 0.6% (SD 8.2%). It is possible that a combination of effective motion correction and automated tumour delineation could improve this. However, improved margin delineation would only be expected to produce further improvements in ADC reproducibility above the 2.7% that we have achieved here.

We applied a 3-parameter model, which includes terms for systematic, MR system related, errors; fitting errors in the ADC estimation and statistical errors arising from inaccuracies in estimating mean ADC. In data where there was no visible movement artefact the largest source of predictable measurement error resulted from differences in the standard error on the mean, estimated from ADC histograms. Consequently, statistical measurement error was much larger in smaller tumours. When the ROI volume is larger than approximately 22.5 cm^3^, the benefit of reduced uncertainty with increased tumour volume begins to plateau.

The 95% confidence limit width for ΔADC% in raw data is 21.1% falling to 2.7% when the estimated ADC values were standardised to the estimated statistical measurement uncertainty. The remaining variability between test and retest values can be attributed to a combination of factors not included in the model (e.g. motion, tumour heterogeneity, SNR). Clearly our error model makes an assumption that the original datasets are accurately co-registered and does not account for movement artefact.

When data is standardised against uncertainty in individual repeatability measurements a number of ROIs affected by motion become outliers (Fig. 4) with the ΔADC% of the remaining ROIs lying mostly within 2% of zero bias. It is clear that accurate interpretation of the observed changes must account for or preferably correct for the level of uncertainty in a repeatability measurement for individual tumours. Following this, the contributing effect of respiratory motion to poor reproducibility and false positive results, can be more accurately assessed. In the current dataset 25% of the data was affected by visible motion artefact.

The development of the error model has very significant implications in the interpretation of ADC data, which is likely to be equally true in other anatomical settings. Use of the model either directly, or by estimation of uncertainty from the lookup table (Fig. 5) enables the investigator to understand the expected statistical errors in individual estimates of mean ADC based on the number of voxels in the sample, combined with the standard deviation of the ADC distribution within the ROI. The lookup table can be used directly to assess the likely significance of any change in ADC observed in a single tumour as a result of physiological, pathological or therapeutic response. For group studies, the model may be used to assess reproducibility and therefore significant change thresholds with greater confidence, by standardising the observed data to the level of uncertainty in the measurement. The model may also be used to justify the selection of minimal tumour size in order to minimise measurement uncertainty.

We have presented this work as a proof of concept of the potential benefits for the application of error modelling to improve sensitivity to therapeutic or physiological change. We conducted the study in patients with metastatic colorectal carcinoma where metastases tend to be relatively well delineated and typically show significant signal intensity variation from normal background^38^. Metastatic lesions from other biological sources can show a wide variation in imaging characteristics and signal intensity^39^. Choosing a specific type of tumour metastasis with a relatively consistent imaging morphology limits variability from sources not included in the error model. The conclusions drawn here concerning the impact of tumour volume on measurement accuracy will apply for all types of tumour, although additional errors may be expected in cases where there is biological difficulty with ROI delineation. It will be important for individual studies to assess interobserver variability in ROI delineation and to develop reproducible manual, automated or semi-automated methods for detection of tumour margins. Similarly, alterations in local tissue vascularisation or peri-tumoural tissue density may affect the reproducibility of ROI delineation in individual cases or following therapy, affecting sensitivity to changes in tumoural ADC. Appropriate error modelling using the techniques described here can still be expected to deliver similar improvements in sensitivity.

The modelling techniques described here are applied directly to calculated parametric images. Sources of error in the calculation of the parametric image are not addressed and cannot be addressed in this methodological approach. There is therefore a clear need to perform quality control which must, in any clinical study, include detection of and correction of physiological or patient motion prior to the calculation of parametric images. In this study we have deliberately excluded tumours from areas where significant motion would be expected in order to provide data to test the error model concept. Despite this, we have identified visible motion in almost 25% of tumours. Most of these were tumours in the sub-phrenic region (Table 2). Errors resulting from failure to correct the motion artefact were ameliorated by the improved statistical power in large tumours but had a very significant detrimental effect on the estimation of ADC reproducibility in smaller lesions. We would assume that in clinical studies appropriate motion correction would be performed or, based on these findings and those of previous studies, that datasets showing significant motion artefact would be excluded. A number of data registration methods to correct for movement during data acquisition, prior to calculation of parametric images, have been described and are readily available^40^.

The findings presented here identify several methodological approaches that are essential to improve sensitivity to therapeutic or physiological change. Firstly; it is essential that extraneous motion be identified and corrected prior to calculation of parametric images. Any error introduced by motion cannot be addressed or corrected following the calculation of the ADC map. Secondly; significant reductions in sensitivity to change are associated with smaller tumours. These effects are significant below a tumour size of approximately 22.5 cm³. Clearly exclusion of tumours below this size is undesirable and impractical in most clinical applications. It is impossible to identify a single "cut-off" volume that should be applied across clinical studies. If the expected magnitude of change in ADC is known, or where a threshold for detection sensitivity is desired then the use of the error model can provide a recommended minimum tumour volume for individual studies. This can be approximated by use of the lookup table provided in Fig. 5. This approach should be used to identify inclusion/exclusion criteria for individual studies. Thirdly; application of the error model within a clinical study will allow significant reductions in minimum tumour size due to the consequent improvements in sensitivity for detection of change in ADC.

Our study has a number of limitations. The multi-site nature of the design meant that each site had to follow a standardised protocol that may not represent the optimal results available from individual manufacturers system. We did not attempt to quantify inter observer reliability which is likely to be another source of variability in future study designs. We have not attempted to correct for the clear visible motion artefact in a subset of the patients, which we have shown to be a significant contribution to reduced accuracy in estimates of ADC. This reflects the lack of an effective motion correction technique, which must form a priority for subsequent methodological research in this area.

## Conclusion

We have presented a model that describes statistical sources of variation, and illustrate how this can be used to determine the level of uncertainty in a repeatability measurement of ADC for an individual tumour based on the ROI size and the standard deviation of the ADC distribution. We have standardised observed data to their level of uncertainty, a method that can be used for group studies, to estimate with more accuracy the confidence limits (95% confidence interval widths) that would determine a statistically significant change in ADC. For small tumour volumes with a wide ADC range of distribution, measurements are likely to have a high degree of uncertainty. A strategy of minimum tumour size could optimise statistical power from group studies. For individual tumour assessment, a higher threshold is required in the interpretation of ΔADC%, in order to overcome uncertainty in the measurement. We provide a lookup chart to allow investigators to estimate uncertainty due to statistical error, for any given tumour volume and distribution. Finally, we have also demonstrated that movement artefact is a major remaining source of error suggesting that our technique should be combined with appropriate motion correction strategies, particularly for small tumours^40^.

## Electronic supplementary material

Supplementary material
