# Supplementary material for: A data-driven statistical model that estimates measurement uncertainty improves interpretation of ADC reproducibility: a multi-site study of liver metastases
Source: Sci Rep. 2017 Oct 26;7:14084. doi: 10.1038/s41598-017-14625-0 (PMC5658431; doi:10.1038/s41598-017-14625-0)
Supplement: Supplementary file 1 — Supplementary material [file 41598_2017_14625_MOESM1_ESM.pdf]

A data-driven statistical model that estimates measurement uncertainty improves interpretation of ADC reproducibility: a multi-site study of liver metastases

*Ryan Pathak [1], Hossein Ragheb [1], Neil A Thacker [1], David M Morris [1], Houshang Amiri [2], Joost Kuijer [3], Nandita M deSouza [4], Arend Heerschap [2], Alan Jackson [1]

1. University of Manchester, Wolfson Molecular Imaging Centre, Manchester, Lancashire, UK.
2. Radboudumc, Radiology and Nuclear Medicine, Nijmegen, Gelderland, NL
3. VU University Medical Center, Physics & Medical Technology, PO Box 7057 Amsterdam, NL 1007MB
4. Institute of Cancer Research, MRI Unit, Downs Road, Sutton, Surrey, UK SM2 5PT

Supplementary Information

Appendix 1

The ADC is the decay parameter from an exponential fit of the loss in signal intensity at a given pixel location, between the increasingly diffusion sensitive images (b-100, 500, 900 s/mm^2). In clinical data, as the noise distribution is skewed towards the higher b-values, we find that a first order bias correction factor (α) improves the quality of fit as it removes the SNR dependent bias.
We estimate ADC (referred to as *D*), through a likelihood-based parameter optimization, log *P*(*I**D*, *S*0) (the probability of the image data given the assumed parameters)

$$\log P\left(I \mid D, S0\right) = \frac{-1}{2\sigma^2} \sum_{b_k} [I(b_k) - f(b_k, D, S0)]^2 + const \qquad (1.1)$$

where $f(b_k, D, S0)]^2$ is the theoretical value of the bias corrected exponential function and $I(b_k)$ is the signal value from the b-value image pixel. f is a function of b-value

b_k and the current estimates of ADC, D, and no-diffusion signal, $S0$ (at $b_k = 0$). This is computed using

$$f^2(b_k, D, S0) = S0^2 \exp(-2b_k D) + \alpha\sigma_1^2; k \in [1,2,3] \qquad (1.2)$$

Where $k \in [1,2,3]$ refers to the three b-values b_k used, e.g. 100, 500 and 900 s/mm^2. The signal value for no diffusion (b=0) $S0$ is the second parameter that is estimated. α is a fixed value defining the amount of bias correction applied and it may be adjusted depending on the amount of image smoothing corresponding to the specific imaging protocol used by the scanner (for our data α was set to the theoretical value of one). An estimate of the standard deviation (SD) of noise in the image α_I is computed from the distribution of second derivatives (for x and y) around zero, in a central rectangular region on the tissue (http://www.tina-vision.net/docs/memos/2008-010.pdf).

Appendix 2

In order to study a change in ADC or reproducibility, we can use the percentage change in mean ADC as given by

$$R_{12} = 2\frac{(D_1 - D_2)}{(D_1 + D_2)} \times 100 \qquad (2.1)$$

From a basic statistical level, the wider the distribution of values in the ADC histogram used to determine the mean ADC ($(D_1 \text{ or } D_2)$), the larger the standard error of the mean will be and conversely, the larger the sample size (N) the smaller the standard error of the mean will be.

$$\text{Standard Error } (D) = \frac{SD}{\sqrt{N}} \qquad (2.2)$$

However, we would expect the variable R_{12} to be affected by variations in SNR, imaging artifact and motion, to differing degrees between test and retest.
Contributions to errors on R_{12}, defined as $\varepsilon_{R_{12}}$, can be estimated from error propagation based upon the expected errors ($\sigma_{D_1}, \sigma_{D_2}$) on each baseline ADC measurement, D_1 and D_2. We assume that

$$\sigma_{D_i} = \frac{SD\left(D_i\right)}{\sqrt{N'}} \tag{2.3}$$

Where σ_{D_i} is a measure of the width of the ADC distribution and N' is the number of independent measurements in the region.
For mean parameters x and y, with measurement errors in each case defined by (1.5), we wish to find the measurement error on parameter z, defined as

$$z = 200\frac{\left(x-y\right)}{\left(x+y\right)} \tag{2.4}$$

Using error propagation, the measurement error on z is given by

$$\varepsilon_z^2 = \sigma_x^2\left(\frac{dz}{dx}\right)^2 + \sigma_y^2\left(\frac{dz}{dy}\right)^2 \tag{2.5}$$

The derivatives of z with respect to x and y are

$$\frac{dz}{dx} = \frac{400y}{\left(x+y\right)^2}; \frac{dz}{dy} = \frac{-400x}{\left(x+y\right)^2} \tag{2.6}$$

Hence, we can write

$$\varepsilon_z = \frac{400}{\left(x+y\right)^2}\sqrt{y^2\sigma^2 + x^2\sigma_y^2} \tag{2.7}$$

Contributions to errors on R_{12}, defined as $\varepsilon_{R_{12}}$, can therefore be estimated from

$$\varepsilon_{R_{12}}\left(\sigma_{D1},\sigma_{D2}\right)=\frac{400\sqrt{D_1^2\sigma_{D2}^2+D_2^2\sigma_{D1}^2}}{\left(D_1+D_2\right)^2} \tag{2.8}$$

Appendix 3

In order to construct likelihood for multiple repeat samples (N), based on the difference between the expected and observed variances, we assume an approximate Gaussian distribution for the difference $y_{1i}-y_{2i}$

$$\log L=\sum_i^N\frac{\left(y_{1i}-y_{2i}\right)^2}{\sigma_{yi}^2}+\log\left(\sqrt{2\pi\sigma_{yi}}\right) \tag{3.1}$$

The last term is normally omitted from fitting routines (on the basis that is a constant), but is needed if the value of σ_{yi} is allowed to vary as part of the fit (as is the case here).

The resulting log likelihood cannot be treated as a χ^2 statistic as the fitting process guarantees that χ^2 = N. However, once the error model is determined we can assess the adequacy of the model for describing sub groups *g*, with

$$\chi^2=\sum_{i\in g}^N\frac{\left(y_{1i}-y_{2i}\right)^2}{\sigma_{yi}^2} \tag{3.2}$$

The three parameters we have used to describe observed reproducibility are obtained by fitting the datasets without visible GM, resulting in the error formula

$$\varepsilon_{R_{12}}^2=\beta^2\varepsilon_{R_{12}}^2\left(\sigma_{D_1},\sigma_{D_2}\right)+\varepsilon_{R_{12}}^2\left(\sigma_{fix},\sigma_{fix}\right)+\varepsilon_{sys}^2 \tag{3.3}$$

Having a distribution $\left(\frac{R_{12}[j]}{\varepsilon_{R_{12}[j]}}\right)$, with unit standard deviation. We then minimize the likelihood cost function

$$-\log L = \sum_{j=1}^{J}\left[\frac{1}{2}\left(\frac{R_{12}[j]}{\varepsilon_{R_{12}[j]}}\right)^{2} + \log\left(\sqrt{2\pi\varepsilon_{R_{12}[j]}}\right)\right] \qquad (3.4)$$

in order to estimate the three parameters (β, σ_{fix}, ε_{sys}) where j is the sample number for the whole population J (in this case J = 60, i.e. 15 out of 20 data sets without visible motion artifact, multiplied by the 4 alternative ROI methods described in the methods section).
